# Fighting Social Isolation With Nostalgia: Nostalgia as a Resource for Feeling Connected and Appreciated and Instilling Optimism and Vitality During the COVID-19 Pandemic

**DOI:** 10.3389/fpsyg.2021.740247

**Published:** 2021-11-18

**Authors:** Rogelio Puente-Díaz, Judith Cavazos-Arroyo

**Affiliations:** ^1^School of Business and Economics, Universidad Anáhuac México, Mexico City, Mexico; ^2^Centro Interdisciplinario de Posgrados, Universidad Popular Autónoma del Estado de Puebla, Puebla, Mexico

**Keywords:** nostalgia, COVI-19 pandemic, social isolation, autobiographical memory, social goal

## Abstract

In two experiments, we tested the influence of bringing to mind a memory of a special moment versus an ordinary moment on nostalgia and whether this elicited nostalgia was related directly to gratitude and the satisfaction of need for relatedness and indirectly to optimism and vitality. Participants from Mexico were first asked to state how the pandemic of COVID-19 has affected their lives. After, participants were randomly assigned to one of two conditions: Memory of special moment versus memory of ordinary recent moment (study 1) or memory of special moment versus or memory of ordinary moment from the same life period as the special moment (study 2). After, participants completed a battery of questionnaires assessing nostalgia, gratitude and optimism (study 1) or nostalgia, satisfaction of need for relatedness, and vitality (study 2). Results from study 1 showed a positive influence of bringing to mind a special moment on nostalgia. Nostalgia was positively related to gratitude, which was then related positively to optimism. Similarly, results from study 2 showed a positive influence of bringing to mind a special moment on nostalgia. Nostalgia was positively related to satisfaction of need for relatedness, which then had a positive relationship with vitality. In both studies, the indirect sequential effect of bringing to mind a special moment on optimism and vitality was significant.

## Introduction

As we continue experiencing one of the worst health crises ever seen, individuals complain about social isolation, anxiety, stress, worry, and insomnia due to prolonged periods of confinement (Bland et al., 2021; Panchal et al., 2021). While we do not deny the importance of providing long term solutions to these problems and challenges, we should not underestimate the power of cognitive processes such as recalling important life events to help individuals cope with some aspects of the current situation. Specifically, we are interested in examining how feelings of nostalgia defined as “a longing, usually sentimental, to experience again some real or imagined former pleasure” (Webster’s Dictionary, 1989, pp. 685), could help individual feel connected and appreciated, eliciting optimism and vitality. Nostalgia is considered a coping resource to deal with social threats (Sedikides and Wildschut, 2020) and the current pandemic poses different kinds of social threats to many individuals.

We take a situated cognition approach in which the information that is brought to mind is capable of influencing cognitive processing (Schwarz, 2010), including the recall of important moments and the experience of feelings of nostalgia. In addition, models of situated cognition suggest that emotions represent an interaction with the social world (Mesquita, 2010). Given that thinking is for doing, we suggest that recalling important life events could be a powerful resource to meet socials goals and feel more optimistic about the future and with more vitality under the current challenges posed by the pandemic.

We posit the following modest, incremental contributions of our study: (1) It is important to test the applied implications of what we have learnt about autobiographical memory and nostalgia in the past decade or so and how cognition and emotion interact under real social threats (see Bland et al., 2021 for a recent study; Sedikides and Wildschut, 2020), specially with less extensively-studied countries such as Mexico (Hepper et al., 2014). (2) Social threats have often been created in laboratory settings. Under the current pandemic, we have the opportunity to use a real social threat that most individuals could relate to, even though there might be differences in how individuals appraise the current situation. (3) We seek to answer a recent call for more research testing the implications of positive psychology under the current pandemic (Waters et al., 2021).

Consequently, we test, in two experiments, the influence of bringing to mind special moments, as opposed to ordinary moments, on nostalgia and whether this elicited nostalgia is capable of relating positively to feeling grateful and connected to others. In addition, we also examine the relationships between feeling grateful and connected to others and optimism and vitality. In order to reach our research goals, we briefly review the relevant literature and propose some research hypotheses.

### Social Context

One of the natural consequences of lockdowns is social isolation (Panchal et al., 2021), where individuals are unable to achieve their social goals. College students were sent home on March of 2020 by the Mexican government to try to reduce the number of people infected with COVID-19. During data collection and until August of 2021, students had not returned to their universities. Consequently, students spent more than 17 months taking classes online until some universities reopened in the middle of August of 2021. Consequently, it is safe to say that most students experienced threats to the self.

### Nostalgia

Nostalgia represents a coping mechanism that individuals could turn to when feeling lonely, isolated, stressed out or meaningless (Sedikides et al., 2015; Sedikides and Wildschut, 2018). The problem is that, naturally, individuals spend more time thinking about the future than the past (Baumeister et al., 2016). Empirical findings on nostalgia suggest that experiencing nostalgia by recalling important life events could help individuals achieve their social goals indirectly (Juhl et al., 2021). Similarly, conceptual developments on the functions of autobiographical also indicate the achievement of social goals as an important function (Harris et al., 2014). This positive effect of nostalgia has been well-documented across several studies (Cheung et al., 2013, 2016; Hepper et al., 2021; Jiang et al., 2021). Given the social threats experienced by many individuals (Panchal et al., 2021), including college students in Mexico, we posit that this social context represents a research opportunity to examine how remembering special moments from the past can help cope momentarily with some of the threats to the self, experienced by many and help individuals see a brighter future. Looking at our COVID-19 free past might help shed light onto our COVID-19 relatively free future (Sedikides and Wildschut, 2020).

As documented extensively (Sedikides et al., 2015; Sedikides and Wildschut, 2020), the main source of nostalgia is the recollection of important life events. The recollection of mundane daily events does not lead to the same positive outcomes as the recollection of important moments (Newman et al., 2020). These special memories tend to be more positive than negative, involving interaction with others, in which the self, as the protagonist, transports itself back to those good all times (Evans et al., 2021). Instead of using the standard event reflection task, in which participants are asked to bring to mind a nostalgic event (Sedikides et al., 2015), we asked participants to recall an important life event, without requesting a nostalgic event, from the past to test the following hypothesis:

H1: Participants asked to bring to mind the memory of an important moment from their past would experience higher levels of nostalgia than participants asked to bring to mind an ordinary recent moment from the previous week (study 1) or an ordinary moment from their past (study 2).

### Gratitude and Need for Relatedness

Two consequences of nostalgia are its relational outcomes and motivational potency. Regarding its relational outcomes, nostalgia often leads to feeling connected to close others and appreciated (Cheung et al., 2013; Juhl et al., 2021). These feelings of connection and appreciation are usually accompanied by motivational outcomes such as optimism and approach motivation, among others (Stephan et al., 2014; Sedikides and Wildschut, 2020). The self-regulatory model of nostalgia suggests that when the self feels threatened, nostalgia is a resource that helps restore balance by given meaning to life and helping individuals look forward to the future (Stephan et al., 2014). Hence, we use this model to examine first how nostalgia leads to feeling connected and grateful in the form of the satisfaction of the need for relatedness (Ryan and Deci, 2017) and a social emotion such as gratitude (Smith et al., 2017). Two studies showed that the “causal link” going from social outcomes to motivational outcomes provided a better fit than the sequence of motivational to social outcomes (Cheung et al., 2013, 2016). Hence, we posit first a connection between nostalgia and social outcomes. To our knowledge, this is first study to test a connection between nostalgia and the satisfaction of the need for relatedness. Conversely, the connection between nostalgia and gratitude has been explored before (van Tilburg et al., 2018). Specifically, one study found that recalling grateful moments could also elicit nostalgia (van Tilburg et al., 2018), suggesting communalities between both emotions. Similarly, another study found a moderate, positive zero-order correlation between gratitude and nostalgia and some similarities in their appraisal profile (van Tilburg et al., 2019). Both studies identified the emotional profiles of gratitude and nostalgia, without paying special attention of how nostalgia might relate to gratitude under a social threat. Consequently, we test the following two hypotheses:

H2: The feelings of nostalgia elicited by recalling significant moments from the past would have a positive relationship with a social emotion such as gratitude (study 1).

H3: The feelings of nostalgia elicited by recalling important moments from the past would have a positive relationship with the satisfaction of the need for relatedness (study 2).

### Optimism and Vitality

**FIGURE 1:**
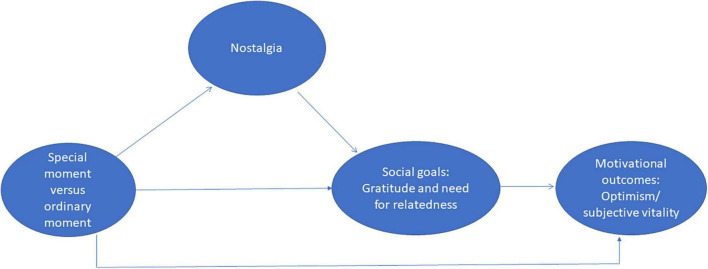
Conceptual model.

Motivation is particularly relevant given the threats to the self, posed by the pandemic. Hence, we examine two motivational consequences in the form of optimism (Carver and Scheier, 2001) and subjective vitality (Ryan and Frederick, 1997). The core characteristic of optimism is expecting good things in the future (Carver and Scheier, 2001). Similarly, vitality is characterized by feeling alive, alert, and with energy, which are crucial to enjoy the present and plan for the future (Ryan and Deci, 2001; Sedikides et al., 2016; See Figure 1 for conceptual model). Previous studies have shown a positive relationship between nostalgia and optimism (Cheung et al., 2013, 2016) and between nostalgia and vitality (Sedikides et al., 2016). Yet, these studies did not use a social threat or conducted their studies with less extensively-studied countries. Hence, we replicate the examination of the following hypotheses:

H4: Feelings of gratitude would have a positive relationship with optimism.

H4b: There would be a sequential positive indirect effect of recalling important events on optimism *via* its influence on nostalgia and the relationship of nostalgia with gratitude.

H5: The satisfaction of need for relatedness would have a positive relationship with subjective vitality.

H5b: There would be a sequential positive indirect effect of recalling important events on subjective vitality *via* its influence on nostalgia and the relationship of nostalgia with the satisfaction of the need for relatedness.

In sum, the purpose of the present investigation is twofold. First, we test the influence of recalling important events on nostalgia. Second, we examine how nostalgia relates to feeling connected with important others and feeling grateful and how these two positive outcomes might be connected positively with optimism and subjective vitality under the difficult conditions of prolonged lockdowns. Our basic premise is that nostalgia helps achieve social goals indirectly, which then facilitates seeing a brighter future.

## Overview of Studies

In experiment one, we tested the influence of bringing to mind the memory of a special moment versus the memory of an ordinary moment from the previous week on nostalgia and then the associations between the elicited nostalgia, gratitude and optimism. In experiment two, we conducted a conceptual replication and examined the influence of bringing to mind the memory of a special moment versus the memory from an ordinary moment on nostalgia and then the associations between the elicited nostalgia and the satisfaction of the need for relatedness and subjective vitality. After manipulating type of memory, we did not manipulate but only measured, the mediator, social and motivational outcomes. Consequently, we cannot provide empirical evidence in favor of our causal order, as suggested by methodological scholars (Spencer et al., 2005). While we acknowledge this limitation, our thinking is that it might not be necessary to manipulate additional variables because the relationship between state or elicited nostalgia and social outcomes is well established (Hepper et al., 2012; Cheung et al., 2013, 2016; Lasaleta et al., 2014; Reid et al., 2015; Sedikides et al., 2016; Abeyta et al., 2020; and at least 5 or 6 additional studies). Similarly, while it is recommended to test experimentally the influence of social outcomes on motivational outcomes, the relationship has been well-established empirically as well (see Routledge et al., 2011; Cheung et al., 2013, 2016; Lasaleta et al., 2014; and others) and some studies have tested alternative models, from motivational to social outcomes, without obtaining positive results (Cheung et al., 2013, 2016). We decided then not to conduct additional experiments and examined only the relationships between social and motivational outcomes. We did test alternative models in which the causal order was reserved, from motivational to social outcomes, and reported the results.

We conducted a power analysis for study 1 to guide our sample size decisions for both studies. After, we set the length of data collection to 8 weeks, seeking to at least achieve the sample size calculated in our power analysis. Both studies received approval from an internal human subjects committee. In addition, all questionnaires were administered in Spanish.

## Method of Study 1

### Participants

We conducted a power analysis with G^∗^Power (Faul et al., 2007) to determine the number of participants needed for a power of 0.95 with an effect size of 0.25 and two groups. From the empirical literature on nostalgia, we observed that the effect sizes were small to medium (e.g., Sedikides et al., 2015; Sedikides and Wildschut, 2020). This is why we used a value of 0.25 to calculate the sample size needed. Results showed that we needed a sample size of at least 210. Hence, we decided to collect data for 8 weeks seeking reach our goals of having at least 210 participants. After 8 weeks of data collection, participants were 332 (66% women and 34% men; 97% were in the 18–25 age range) college students from two universities in Mexico. The data was collected in the fall of 2020 online using Google forms. All participants stopped attending school on March 21st of 2020 due to the restrictions set by the Mexican government and they did not go back until August of 2021 (17 months).

### Procedure

We first asked participants from to tell us how the pandemic has affected their lives. This open-ended question served two important purposes. First, it allowed us to validate whether the pandemic has represented a threat to the self. Second, it allowed to contextualize our investigation, given that participants were probably not always thinking about the pandemic and its effects, consistent with models of situated cognition (Schwarz, 2010). This contextualization helped test with more confidence the potential implications of special memories and nostalgia for social and motivational outcomes. It helped us test how the past can help see a brighter future under difficult conditions.

After, participants were randomly assigned to one of two conditions: special moment from their years in high school or ordinary moment from the previous week. Participants read the following instructions taken from the relevant literature on nostalgia (e.g., Sedikides et al., 2015):

Special moment: For this activity, we would like you to think about your last year in high school. Within this year, we would like you to find a special moment that you personally experienced, a moment that represents a significant memory for you. Please, describe this moment in the space below with as many details as possible.

Recent ordinary moment: For this activity, we would like you to think about last week. Within this week, we would like you to find an ordinary moment that you personally experienced, a moment that represents an ordinary memory for you. Please, describe this moment in the space below with as many details as possible.

### Measures

#### Nostalgia and Gratitude

We assessed all items by using the following prompt: Remembering this special (ordinary) moment makes me feel? In a scale from “I do not feel like that at all” (1) to “I completely feel like that” (10). The items for nostalgia were: nostalgic, happy but longing to relive those types of moments, with nostalgic feelings, with the sensation that I miss those moments (α = 0.92), taken from a review article on nostalgia (Sedikides et al., 2015). The items for gratitude were: grateful, understood, and appreciated (α = 0.78), taken from the relevant literature (Frias et al., 2011). Given that nostalgia and gratitude were assessed under the same prompt, we conducted a Confirmatory Factor Analysis to examine divergent validity. Results for the measurement model of a two-factor solution showed an acceptable model fit χ^2^ = 45.21, *p* < 0.001, (df = 12), RMSEA = 0.09, CFI = 0.99 and TLI = 0.99. Examination of the factor loadings showed that they were all significant and in the expected direction (ranging from 0.79 to 0.94). The bivariate latent correlation was 0.76, below the recommended limit of 0.85 (Brown, 2006).

#### Optimism

This is a four-item measure designed to assess optimism (Cheung et al., 2016). The instructions asked participants to pay attention to their current sensations and used a scale from 1 (I do not feel that at all) to 10 (I completely feel like that) to answer all questions. We used the total score from all four items, which showed acceptable levels of internal consistency (α = 0.76).

## Results of Study 1

Participants reported a wide variety of negative consequences from the pandemic. Specifically, we focused on coding any answers that would fall into the following categories: social isolation, negative affect such as anxiety, stress, worry, sadness, depression, and reduced energy, death of a love one, and difficulty sleeping. Results showed that 88% of the participants reported at least one indicator of said categories, lending evidence to the idea that the pandemic represented a threat to the self. In addition, over 40% of participants reported economic challenges such as unemployment, difficulty finding a job, job insecurity, and reduced income. Hence, our assumption that participants were facing significant challenges due to the prolonged lockdowns was supported. In addition, participants brought to mind their current struggles before being assigned to one of two experimental conditions. This procedure tried to resemble how natural recalling might take place during lockdowns.

We conducted a one way between subjects Analysis of Variance (ANOVA) to estimate the effect of the experimental condition, memory of special versus ordinary moment, on nostalgia. Results for nostalgia showed a significant effect of the experimental condition, *F*(1, 331) = 109.84, *p* < 0.001. Specifically, recalling a special moment led to higher levels of nostalgia, Mspecial = 8.84, SD = 1.40 than recalling an ordinary moment, Mordinary = 6.27, SD = 2.82, Cohen’s δ = 1.15. Similarly, the effect on gratitude was also significant, *F*(1, 331) = 47.38, *p* < 0.001, Cohen’s δ = 0.76. Last, the effect on optimism was not significant, *F*(1, 331) = 3.03, *p* = 0.08, Cohen’s δ = 0.20.

### Mediation and Sequential Model

While we could test a model in which gratitude mediates the relationship between the experimental condition and nostalgia, research on appraisal and emotions has shown that one of the defining features of nostalgia is its greater temporal distance, which is not shared with gratitude (van Tilburg et al., 2019). We followed the guidelines set by Hayes (2018) to test our mediation model and the sequential effect of recalling a special moment from high school versus recalling an ordinary moment from the previous week. A bootstrap test (PROCESS, model 4, Hayes, 2018) showed that in the mediation model, there was a significant effect of the experimental condition on nostalgia, *b* = 2.56 *p* < 0. 001. The relationship between nostalgia and gratitude was significant, while controlling for the influence of the experimental condition, *b* = 0.49, *p* < 0.001. Conversely, the influence of the experimental condition was not significant, *b* = 0.18, *p* = 0.36. Last, the indirect effect of the experimental condition on gratitude was significant, 1.25, CI = 0.89, 1.62. The effect size was 0.38, CI = 0.26, 0.50. Hence, our results showed that bringing to mind a special moment from high school led indirectly to feeling grateful and appreciated, in the form of gratitude, through its influence on nostalgia. In addition, a bootstrap test (PROCESS, model 6, Hayes, 2018) showed a significant sequential indirect effect of the experimental condition on optimism, 0.33, CI = 0.17, 0.56., *via* its influence nostalgia and the relationship between nostalgia and gratitude. The effect size was 0.24, CI = 0.12, 0.37, which could be interpreted as a quarter standard deviation gain in optimism when recalling a special moment versus an ordinary moment. Hence, participants felt more optimistic after bringing to mind a significant memory from their years in high school.

### Alternative Sequential Model

Feelings of nostalgia were elicited by the experimental condition, consequently, the position of nostalgia was fixed. Yet, we could test a model in which optimism precedes gratitude. A bootstrap test (PROCESS, model 6, Hayes, 2018) showed that the alternative sequential indirect effect of the experimental condition on gratitude was not significant, 0.03, CI = −0.03, 0.09., *via* its influence nostalgia and the relationship of nostalgia with optimism.

### Brief Discussion

Our results were consistent with the vast literature on nostalgia showing the relational and motivational consequences of nostalgia (Sedikides et al., 2015; Sedikides and Wildschut, 2018, 2020). Participants asked to recall a special moment from their high school years reported higher levels of nostalgia, which was then positively associated with gratitude. Participants were then able to achieve their social goals briefly. In addition, gratitude had a positive relationship with optimism. In order to increase the validity of our results, we conducted a conceptual replication by asking participants to recall a special moment from their years in junior high or an ordinary event from the same life period to address the limitation of using an event from the previous week as the control condition in study 1. In addition, we extended our examination of relational goals by assessing the satisfaction of the need for relatedness and added one motivational outcome in the form of vitality as two potential consequences of nostalgia.

## Methods of Study 2

### Participants

For our second study, we collected data for 8 weeks, seeking to have at least 210 participants. After 8 weeks of data collection, participants were 477 (65% women and 35% men; 93% were in the 18–25 age range) college students from two universities in Mexico. The data was collected online in the spring of 2021.

### Procedure

As in study 1, participants first reported how the pandemic has affected their lives. After, participants were then randomly assigned to one of two conditions: special moment from their years in junior high or ordinary moment from the same life period. Participants read the following instructions taken from the relevant literature on nostalgia (e.g., Sedikides et al., 2015):

Special moment: The instructions were the same as study 1 with the only difference that we asked participants to recall a moment from their years in junior high.

Ordinary moment: The instructions were similar to study 1, but asked participants to recall an ordinary moment from their years in junior high.

### Measures

The same nostalgia items as study 1 were used. The levels of internal consistency were acceptable (α = 0.93).

#### The Basic Psychological Need Satisfaction and Frustration Scale (BPNSFS)

This scale uses 24-items to measure need satisfaction and frustration for competence, relatedness and autonomy (Chen et al., 2015). We were interested in assessing the satisfaction of the needs of relatedness, hence we only used the four items design to do so with a scale ranging from “I do not feel like that at all” (1) to “I completely feel like that” (10). The items were: I feel that the people I care about, they care about me, connected with the people I care about and they care about me, close and connected with the people that are important to me, and with a sensation of warmness when I am with the people I interact with. The items were answered using the prompt: Remembering this special (ordinary) moment makes me feel? The scores showed acceptable levels of internal consistency (α = 0.93). As in study 1, we conducted a CFA to assess the discriminant validity between the nostalgia and need for relatedness scores given that they were measured with the same prompt. Results for the measurement model showed an acceptable model fit χ^2^ = 59.24, *p* < 0.001, (df = 18), RMSEA = 0.07, CFI = 0.99 and TLI = 0.99. Examination of the factor loadings showed that they were all significant and in the expected direction (ranging from 0.83 to 0.95). The bivariate latent correlation was 0.47, below the recommended limit of 0.85 (Brown, 2006).

#### Subjective Vitality

This scale uses seven items to assess subjective vitality (Ryan and Frederick, 1997). We instructed participant to think about their current feelings and sensations and used a scale ranging from “I do not feel like that at all” (1) to “I completely feel like that” (10). The scores showed acceptable levels of internal consistency (α = 0.85).

## Results of Study 2

Consistent with study 1, 92% of participants reported at least one indicator of the following categories: social isolation, negative affect, death of a love one, and difficulty sleeping. The observed increase, from 88 to 92% was probably due to the fact that the second data collection took place after a longer period of confinement. In addition, over 50% of participants reported economic challenges such as unemployment, difficulty finding a job, job insecurity, and reduced income. Hence, our assumption that this pandemic has posed threats to the self was supported.

As in study 1, we conducted a one way between subjects Analysis of Variance (ANOVA) to estimate the effect of the experimental condition, memory of special versus ordinary moment, on nostalgia. Results for nostalgia showed a significant effect of the experimental condition, *F*(1, 477) = 6.57, *p* = 0.01. Specifically, recalling a special moment led to higher levels of nostalgia, Mspecial = 7.78, SD = 2.19 than recalling an ordinary moment, Mordinary = 7.19, SD = 2.79, Cohen’s δ = 0.24. Conversely, the effects on need for relatedness or vitality were not significant, *F*(1, 477) = 2.60, Cohen’s δ = 0.14, *p* = 0.11; *F*(1, 477) < 1, Cohen’s δ = 0.09, respectively.

### Mediation and Sequential Model

**FIGURE 2:**
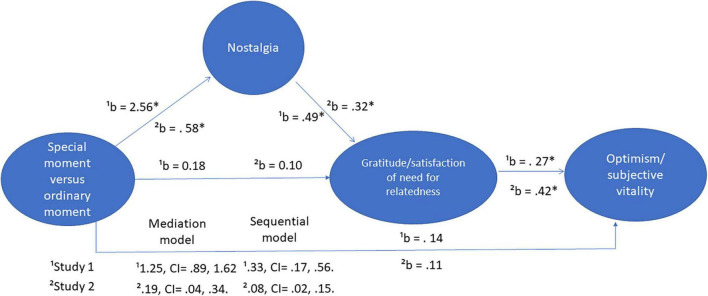
Summary of results.

As in study 1, we followed the guidelines set by Hayes (2018) to test our mediation model and the sequential effect of recalling a special moment from high school versus recalling an ordinary moment from the same period of life. A bootstrap test (PROCESS, model 4, Hayes, 2018) showed that in the mediation model, there was a significant effect of the experimental condition on nostalgia, *b* = 0.58, *p* = 0.01. The relationship between nostalgia and need for relatedness was significant, while controlling for the influence of the experimental condition, *b* = 0.32, *p* < 0.001. Conversely, the influence of the experimental condition was not significant, *b* = 0.10, *p* = 0.54. Last, the indirect effect of the experimental condition on need for relatedness was significant, 0.19, CI = 0.04, 0.34. The effect size was 0.10, CI = 0.02, 0.17. Hence, our results showed that bringing to mind a special moment from high school led indirectly to the satisfaction of the need for relatedness, through its influence on nostalgia. In addition, a bootstrap test (PROCESS, model 6, Hayes, 2018) showed a significant sequential indirect effect of the experimental condition on vitality, 0.08, CI = 0.02, 0.15., *via* its influence nostalgia and the relationship of nostalgia with the satisfaction of the need for relatedness. The effect size was 0.04, CI = 0.01, 0.08, which could be interpreted as a five percent of a standard deviation gain in vitality when recalling a special moment versus an ordinary moment. The observed effect size was smaller than in study 1. Hence, participants felt more vitality after bringing to mind a significant memory from their years in junior high (See Figure 2 for summary of results and Table 1 for descriptive statistics).

**TABLE 1:** Descriptive statistics for study 1 and 2 as a function of the experimental condition.

	**Special moment**		**Ordinary moment**	
	**M**	**SD**	**M**	**SD**
**Experiment 1**				
Nostalgia	8.84	1.40	6.27	2.82
Gratitude	9.14	1.19	7.71	2.39
Optimism	8.85	1.25	8.57	1.57
**Experiment 2**				
Nostalgia	7.78	2.19	7.19	2.79
Need for relatedness	8.63	1.82	8.35	2.04
Vitality	6.97	1.86	6.81	1.72

### Alternative Sequential Model

As in study 1, feelings of nostalgia were elicited by the experimental condition, consequently, the position of nostalgia was fixed. Yet, we could test a model in which vitality precedes the satisfaction of the need for relatedness. A bootstrap test (PROCESS, model 6, Hayes, 2018) showed that the alternative sequential indirect effect of the experimental condition on need for relatedness was not significant, 0.003, CI = −0.01, 0.02, *via* its influence nostalgia and the relationship of nostalgia with vitality.

### Brief Discussion

Results showed additional support for the proposition that recalling important moments led to greater nostalgia, which was then positively associated with relatedness and vitality. The results were consistent with the proposition that turning back to the past helps individuals see a brighter future (Sedikides and Wildschut, 2020). It was also consistent with the idea that nostalgia helps achieve social goals (Juhl et al., 2021). Yet, the effect size of the experimental condition on nostalgia was smaller than the one observed in study 1.

## General Discussion

The purpose of present investigation was to examine the relationships between nostalgia, coming from recalling special life moments, on two relevant relational outcomes and social goals and how these two relational outcomes were associated with optimism and subjective vitality. Six hypotheses were proposed and tested. We found support for all them, yet the effect size of the experimental manipulation in study 2 was smaller. By conducting two experiments, we answered a recent call for more research on positive psychology interventions under the pandemic of COVID-19 (Waters et al., 2021). We discussed the implications of our results.

As suggested by health experts (Panchal et al., 2021), we are living an extremely difficult health crisis in which individuals are experiencing a wide range of psychological symptoms such as anxiety, depression, social isolation, and a sense that life is meaningless. Consequently, individuals are unable to meet some of their social goals. We tested whether bringing a special memory from the past could elicit feelings of nostalgia. Our results showed that this was the case, which was consistent with the vast literature on nostalgia (e.g., Sedikides and Wildschut, 2020). The benefit of recalling a special moment was significantly larger when compared to recalling a recent, ordinary event than when recalling an ordinary event from the same life period. This result could mean that memories from the past are in general more precious than recent memories and that ordinary might feel better now under the current conditions of prolonged lockdowns. Ordinary events might be recalled with a rosier tone (Skowronski et al., 2014). In addition, under prolonged confinements, individuals might come to appreciate ordinary.

The elicited nostalgia was positive related to gratitude and the satisfaction of the need for relatedness. This was consistent with the well-document social benefits of nostalgia (e.g., Juhl et al., 2021). Nostalgia represents an important resource when experiencing threats to the self, due to the lack of social interactions during extensive lockdowns. Nostalgia can help achieve some social goals indirectly (Juhl et al., 2021) by recalling events where the self was a protagonist and important others were involved. In addition, gratitude and satisfaction of the need of relatedness were positively related to optimism and subjective vitality. These results were consistent with the abundant literature on the motivational potency of nostalgia (Sedikides and Wildschut, 2020). More importantly, our results showed that participants who recalled an important moment from their past experienced higher levels of optimism and subjective vitality under difficult conditions, where reporting psychological symptoms have been the norm more than the exception (Panchal et al., 2021). Our results showed that our replication of the examination of the indirect relationships between nostalgia and optimism and vitality obtained similar findings (Cheung et al., 2013, 2016; Sedikides et al., 2016). These results supported the idea that positive psychology interventions might help alleviate momentarily the threats to the self, experienced by many individuals (Waters et al., 2021). The good old times could represent important resources when the self feels threatened.

It is important to note that we only manipulated the recall of a special moment versus an ordinary moment and assessed its influence on nostalgia. Methodological scholars suggest manipulating nostalgia, satisfaction of the need for relatedness or gratitude, mediators, and measured their relationships with optimism and vitality, outcomes, when testing sequential models (Spencer et al., 2005). Future research could try to address these shortcomings.

### Limitations and Future Directions

This study had several limitations. First, we used a sample of convenience, which limits our ability to generalize our results. As shown in our results, our participants did experience economic challenges and threats to the self, coming from the extensive lockdowns, yet these challenges might not be as detrimental as the ones faced by older adults who experienced high unemployment and domestic disputes, among others. Second, our experimental manipulation was brief. Even though we observed positive results, future studies should use longer interventions to examine how and whether nostalgia is a constant resource to achieve social goals and see a brighter future. Third, even though we asked participants how the pandemic has affected their lives in general and almost all answers were negative, indicating the presence of social threats, we did not assess the level of threat and whether individual differences could influence the results. Future studies could start with the same open-ended question, followed by some questions regarding the level of perceived threat. Fourth, even though similar items have been used before to assess state gratitude (Frias et al., 2011), not everyone would agree that feeling appreciated and understood are relevant indicators of state gratitude.

In sum, we found a positive influence of recalling important moments from the past on nostalgia. In addition, nostalgia was positively related to social outcomes in the form of gratitude and satisfaction of the need for relatedness, which were then positively related to optimism and vitality. Hence, during extensive and prolonged lockdowns, nostalgia could be a coping resource that individuals could turn to feel connected to others and see a brighter future.

## Data Availability Statement

The original contributions presented in the study are included in the article/supplementary material, further inquiries can be directed to the corresponding author/s.

## Ethics Statement

The studies involving human participants were reviewed and approved by Universidad Anahuac Mexico. The patients/participants provided their written informed consent to participate in this study.

## Author Contributions

RP-D designed the study, collected the data, analyzed the data, and wrote first and final draft. JC-A designed the study and collected the data and provided critical feedback of the final draft. Both authors contributed to the article and approved the submitted version.

## Conflict of Interest

The authors declare that the research was conducted in the absence of any commercial or financial relationships that could be construed as a potential conflict of interest.

## Publisher’s Note

All claims expressed in this article are solely those of the authors and do not necessarily represent those of their affiliated organizations, or those of the publisher, the editors and the reviewers. Any product that may be evaluated in this article, or claim that may be made by its manufacturer, is not guaranteed or endorsed by the publisher.

## References

[B1] Abeyta A. A. Routledge C. Kaslon S. (2020). Combating loneliness with nostalgia: nostalgic feelings attenuate negative thoughts and motivations associated with loneliness. *Front. Psychol.* 11:1219. 10.3389/fpsyg.2020.01219 32655445 PMC7324708

[B2] Baumeister R. F. Vohs K. D. Oettingen G. (2016). Pragmatic prospection: how and why people think about the future. *Rev. Gen. Psychol.* 20 3–16. 10.1037/gpr0000060

[B3] Bland A. R. Roiser J. P. Mehta M. A. Sahakian B. J. Robbins T. W. Elliott R. (2021). The impact of COVID-19 social isolation on aspects of emotional and social cognition. *Cogn. Emot.* [Online ahead of print]. 10.1080/02699931.2021.1892593 33632068

[B4] Brown T. A. (2006). *Confirmatory Factor Analysis For Applied Research.* New York: Guilford Press.

[B5] Carver C. S. Scheier M. F. (2001). “Optimism, pessimism, and self-regulation,” in *Optimism and Pessimism: Implications For Theory, Research, and Practice* Ed. Chang E. C. (Massachusetts, MA: American Psychological Association), 31–51. 10.1037/10385-002

[B6] Chen B. Vansteenkiste M. Beyers W. Boone L. Deci E. L. Van der Kaap-Deeder J. (2015). Basic psychological need satisfaction, need frustration, and need strength across four cultures. *Motivat. Emot.* 39 216–236. 10.1007/s11031-014-9450-1

[B7] Cheung W. Y. Sedikides C. Wildschut T. (2016). Induced nostalgia increases optimism (via social connectedness and self-esteem) among individuals high, but not low, in trait nostalgia. *Pers. Individ. Differ.* 90 283–288. 10.1016/j.paid.2015.11.028

[B8] Cheung W. Y. Wildschut T. Sedikides C. Hepper E. G. Arndt J. Vingerhoets A. J. J. M. (2013). Back to the future: nostalgia increases optimism. *Pers. Soc. Psychol. Bull.* 39 1484–1496. 10.1177/0146167213499187 23928397

[B9] Evans N. D. Reyes J. Wildschut T. Sedikides C. Fetterman A. K. (2021). Mental transportation mediates nostalgia’s psychological benefits. *Cogn. Emot.* 35 84–95. 10.1080/02699931.2020.1806788 32787551

[B10] Faul F. Erdfelder E. Lang A.-G. Buchner A. (2007). G^∗^Power 3: a flexible statistical power analysis program for the social, behavioral, and biomedical sciences. *Behav. Res. Methods* 39 175–191. 10.3758/BF03193146 17695343

[B11] Frias A. Watkins P. C. Webber A. C. Froh J. J. (2011). Death and gratitude: death reflection enhances gratitude. *J. Posit. Psychol.* 6 154–162. 10.1080/17439760.2011.558848

[B12] Harris C. B. Rasmussen A. S. Berntsen D. (2014). The functions of autobiographical memory: an integrative approach. *Memory* 22 559–581. 23808866 10.1080/09658211.2013.806555

[B13] Hayes A. F. (2018). *introduction To Mediation, Moderation, And Conditional Process Analysis.* New York: The Guilford Press.

[B14] Hepper E. G. Ritchie T. D. Sedikides C. Wildschut T. (2012). Odyssey’s end: lay conceptions of nostalgia reflect its original Homeric meaning. *Emotion* 12 102–119. 10.1037/a0025167 21859192

[B15] Hepper E. G. Wildschut T. Sedikides C. Ritchie T. D. Yung Y.-F. Hansen N. (2014). Pancultural nostalgia: prototypical conceptions across cultures. *Emotion* 14 733–747. 10.1037/a0036790 24866530

[B16] Hepper E. G. Wildschut T. Sedikides C. Robertson S. Routledge C. D. (2021). Time capsule: nostalgia shields psychological wellbeing from limited time horizons. *Emotion* 21 644–664. 10.1037/emo0000728 32191101

[B17] Jiang T. Cheung W.-Y. Wildschut T. Sedikides C. (2021). Nostalgia, reflection, brooding: psychological benefits and autobiographical memory functions. *Conscious. Cogn.* 90:103107. 10.1016/j.concog.2021.103107 33713995

[B18] Juhl J. Wildschut T. Sedikides C. Xiong X. Zhou X. (2021). Nostalgia promotes help seeking by fostering social connectedness. *Emotion* 21 631–643. 10.1037/emo0000720 32297755

[B19] Lasaleta J. D. Sedikides C. Vohs K. D. (2014). Nostalgia weakens the desire for money. *J. Consum. Res.* 41 713–729. 10.1086/677227

[B20] Mesquita M. (2010). “Emoting: A contextualized process,” in *The Mind in Context*, eds Mesquita B. Barrett L. F. Smith E. R. (New York: Guilford), 83–104.

[B21] Newman D. B. Sachs M. E. Stone A. A. Schwarz N. (2020). Nostalgia and well-being in daily life: an ecological validity perspective. *J. Pers. Soc. Psychol.* 18 325–347. 10.1037/pspp0000236 30667254 PMC7513922

[B22] Panchal N. Kamal R. Cox C. Garfield R. (2021). *The Implications of COVID-19 for Mental Health and Substance Use.* Available online at: https://www.kff.org/coronavirus-covid-19/issue-brief/the-implications-of-covid-19-for-mental-health-and-substance-use/ (Accessed March 9, 2021)

[B23] Reid C. A. Green J. D. Wildschut T. Sedikides C. (2015). Scent-evoked nostalgia. *Memory* 23 157–166. 10.1080/09658211.2013.876048 24456210

[B24] Routledge C. Arndt J. Wildschut T. Sedikides C. Hart C. Juhl J. (2011). The past makes the present meaningful: nostalgia as an existential resource. *J. Pers. Soc. Psychol.* 101 638–652. 10.1037/a0024292 21787094

[B25] Ryan R. M. Deci E. L. (2001). On happiness and human potentials: a review of research on hedonic and eudaimonic well-being. *Ann. Rev. Psychol.* 52 141–166. 10.1146/annurev.psych.52.1.141 11148302

[B26] Ryan R. M. Deci E. L. (2017). *Self-Determination Theory: Basic Psychological Needs in Motivation, Development, and Wellness.* New York: The Guilford Press. 10.1521/978.14625/28806

[B27] Ryan R. M. Frederick C. M. (1997). On energy, personality and health: subjective vitality as a dynamic reflection of well-being. *J. Pers.* 65 529–565. 10.1111/j.1467-6494.1997.tb00326.x 9327588

[B28] Schwarz N. (2010). “Meaning in context: Metacognitive experiences,” in *The Mind in Context*, eds Mesquita B. Barrett L. F. Smith E. R. (New York: Guilford), 105–125.

[B29] Sedikides C. Wildschut T. (2018). Finding meaning in nostalgia. *Rev. Gen. Psychol.* 22 48–61. 10.1037/gpr0000109

[B30] Sedikides C. Wildschut T. (2020). The motivational potency of nostalgia: the future is called yesterday. *Adv. Motivat. Sci.* 7 75–111. 10.1016/bs.adms.2019.05.001

[B31] Sedikides C. Wildschut T. Cheung W.-Y. Routledge C. Hepper E. G. Arndt J. (2016). Nostalgia fosters self-continuity: uncovering the mechanism (social connectedness) and the consequence (eudaimonic well-being). *Emotion* 16 524–539. 10.1037/emo0000136 26751632

[B32] Sedikides C. Wildschut T. Routledge C. Arndt J. Hepper E. G. Zhou X. (2015). To nostalgize: mixing memory with affect and desire. *Adv. Exp. Soc. Psychol.* 51 189–273. 10.1016/bs.aesp.2014.10.001

[B33] Skowronski J. J. Walker W. R. Henderson D. X. Bond G. D. (2014). “The fading affect bias: Its history, its implications, and its future,” in *Advances in Experimental Social Psychology*, eds Olson J. M. Zanna M. P. (Cambridge: Elsevier Academic Press), 163–218. 10.1016/B978-0-12-800052-6.00003-2

[B34] Smith A. Pedersen E. J. Forster D. E. McCullough M. E. Lieberman D. (2017). Cooperation: the roles of interpersonal value and gratitude. *Evol. Hum. Behav.* 38 695–703. 10.1016/j.evolhumbehav.2017.08.003

[B35] Spencer S. J. Zanna M. P. Fong G. (2005). Establishing a causal chain: why experiments are often more effective than mediational analyses in examining psychological processes. *J. Pers. Soc. Psychol.* 89 845–851. 10.1037/0022-3514.89.6.845 16393019

[B36] Stephan E. Wildschut T. Sedikides C. Zhou X. He W. Routledge C. (2014). The mnemonic mover: nostalgia regulates avoidance and approach motivation. *Emotion* 14 545–561. 10.1037/a0035673 24708500

[B37] van Tilburg W. A. P. Bruder M. Wildschut T. Sedikides C. Göritz A. S. (2019). An appraisal profile of nostalgia. *Emotion* 19 21–36. 10.1037/emo0000417 29504801

[B38] van Tilburg W. A. P. Wildschut T. Sedikides C. (2018). Nostalgia’s place among self-conscious emotions. *Cogn. Emot.* 32 742–759. 28738756 10.1080/02699931.2017.1351331

[B39] Waters L. Algoe S. B. Dutton J. Emmons R. Fredrickson B. L. Heaphy E. (2021). Positive psychology in a pandemic: buffering, bolstering, and building mental health. *J. Posit. Psychol.* 10.1080/17439760.2021.1871945

[B40] Webster’s Dictionary. (1989). *The New Lexicon Webster’s Dictionary of the English Language.* New York: Lexicon Publications.

